# Effects of hydrological intermittency and anthropogenic pressure on aquatic beetle assemblages in the Martil River (north-western Morocco)

**DOI:** 10.3897/BDJ.14.e192023

**Published:** 2026-07-01

**Authors:** Achraf Guellaf, Nard Bennas, Kawtar Kettani

**Affiliations:** 1 Laboratory of Ecology, Systematics and Conservation of the Biodiversity (LESCB), URL-CNRST N°18, FS, Abdelmalek Essaadi University, Tetouan, Morocco Laboratory of Ecology, Systematics and Conservation of the Biodiversity (LESCB), URL-CNRST N°18, FS, Abdelmalek Essaadi University Tetouan Morocco

**Keywords:** Aquatic beetles, Coleoptera, Mediterranean rivers, Martil Basin, Morocco

## Abstract

This study investigates the spatiotemporal dynamics of aquatic beetle assemblages in the Martil River, northern Morocco, where hydrological intermittency and anthropogenic pressures threaten aquatic diversity and endemism. Over four seasons (spring, summer, autumn 2017, and winter 2018), we sampled 16 sites, grouped into four categories (A–D) along altitudinal and land-use gradients. Our surveys recorded 1,268 individuals representing 50 species from 10 families. Richness and abundance peaked in natural and/or permanent reaches (groups A–B), declining sharply downstream under urbanization and pollution (groups C–D). Gyrinidae dominated numerically (54%), while Dytiscidae exhibited the highest diversity.Adephaga prevailed during dry periods with lentic affinities, in contrast to Polyphaga, which were associated with wet-season lotic conditions. Predators (73%) and swimmers (72%) represented the main functional feeding and locomotion groups. Correspondence analysis revealed seasonal segregation, with rheophilic taxa in wet flows, thermophiles in summer pools, and eurytherms in spring. Co-inertia analysis (CIA) ordination highlighted upstream and midstream refuges with high dissolved oxygen that support sensitive species, in contrast to lowland, slow-flowing streams with elevated BOD_5_, COD, and conductivity, where more tolerant generalist species dominate. Beta-diversity partitioning showed that species turnover (βsim) was the main component of community dissimilarity. PERMANOVA confirmed significant compositional differences between urban and non-urban assemblages. These findings highlight the importance of incorporating permanent mid-reaches into conservation planning and demonstrate the potential of aquatic beetles as indicators of hydrological intermittency.

## Introduction

The Mediterranean Basin stands as a global freshwater biodiversity hotspot, harboring exceptional species richness and endemism despite limited surface area (Abellán et al. 2004, Touaylia 2017, Reid et al. 2019), with systems ranging from perennial rivers to intermittent streams that alternate between flowing and dry phases as groundwater inputs decline (Bonada and Resh 2013).

In these systems, riffle-pool sequences progressively lose riffles as drought advances, leaving isolated pools that persist until floods restore connectivity during wet seasons (Bonada et al. 2006). These hydrological shifts, from perennial connected habitats to intermittent isolated pools, drive macroinvertebrate turnover between desiccation-resistant forms (e.g., desiccation-tolerant eggs) and resilient aerial dispersers, or resilience traits (Strachan et al. 2015).

Water beetles (Coleoptera) stand out among aquatic macroinvertebrates for their exceptional species richness and remarkable functional diversity, which enable them to colonize a wide range of habitats (Ortega et al. 2021). With an estimated 18,000 species worldwide, of which only ~70% have been described, water beetles represent nearly one-third of all aquatic insects and serve as prime indicators of environmental change and freshwater biodiversity assessment (Bennas et al. 2005, Jäch and Balke 2008, Bilton et al. 2019).

Environmental variables (water permanence, flow regime, habitat size, and insularity) shape water beetle assemblages through species sorting, while functional traits enable persistence across hypersaline, eutrophic, lentic, and lotic conditions (Tonkin et al. 2016, Ortega et al. 2021). Yet climate-driven aridification and human pressures confine sensitive and endemic species to fragmented upstream refugia, often within protected areas. Beyond these zones, biotic homogenization accelerates, threatening irreplaceable diversity (Sánchez-Fernández et al. 2004).

Protected areas remain a cornerstone of biodiversity conservation, but their design has historically focused on charismatic terrestrial vertebrates, leaving many freshwater hotspots insufficiently represented (Nogueira et al. 2021). In the Mediterranean region, aquatic beetles have emerged as powerful surrogate taxa for freshwater conservation planning, helping to delineate priority sites and assess the effectiveness of existing protected networks (Abellán et al. 2004, Belhaj et al. 2023a, Sánchez-Fernández et al. 2004).

In northern Morocco, the Rif Mountains form a major aquatic endemism hotspot, where complex relief and biogeographic links to the Iberian Peninsula generate exceptional freshwater diversity from headwaters to coastal wetlands (Benamar et al. 2021, Dakki 1997). Recent analyses reveal peak water beetle species richness in the Rif and Pre-Rif highland regions compared to other areas of Morocco, underscoring their conservation priority (Belhaj et al. 2023b, Benamar et al. 2021, Bennas et al. 2009), thus making their protection even more urgent.

At the national scale, knowledge of aquatic beetle diversity remains incomplete. The distribution of many species is still poorly documented (Belhaj et al. 2023a), and the ecological requirements of endemic and sensitive taxa, particularly their responses to flow intermittency and anthropogenic gradients, remain poorly characterized. These critical gaps in knowledge and taxonomy, combined with IUCN criteria designed primarily for vertebrates (Dakki 1997, Benamar et al. 2021), hamper the effective use of aquatic invertebrates, especially water beetles, in pinpointing key conservation areas or guiding the expansion of protected area networks in northern Morocco. Overcoming these challenges requires intensified research and tailored conservation criteria to enable aquatic insects' integration into freshwater biodiversity protection strategies.

The Martil river basin occupies a strategically important geographical position in the northwestern corner of Morocco along the Mediterranean coast, within the Mediterranean Inter-continental Biosphere Reserve (Guellaf and Kettani 2021a). Over 50% of its territory is protected, yet this basin experiences the combined effects of seasonal hydrological variation, environmental gradients, and intensifying human pressures, particularly agriculture, urbanization, and industry in lowland reaches that profoundly impact aquatic macroinvertebrate assemblages (Guellaf and Kettani 2021b).

By evaluating spatiotemporal dynamics of aquatic beetle assemblages along altitudinal, land-use, and hydrogeological gradients in the Martil River basin, this study aims to identify high-conservation-value reaches outside protected networks and to determine how habitat characteristics and functional traits drive assemblage differentiation.

## Materials and Methods

**Study area**

The Martil River Basin is situated in the northwestern Rif of Morocco, within the Tangier-Tétouan-Al Hoceima region. It covers an area of about 1,259 km² and extends from the Mediterranean coastline toward the rugged interior of the Rif Mountains (Guellaf and Kettani 2025, Taidi et al. 2025), featuring hilly corridors and narrow valleys. Elevations range from sea level to 1,782 m, with a mean altitude of approximately 424 m (Guellaf et al. 2025b).

The basin’s hydrographic network originates in the forested highlands of Jbel Bouhachem and adjacent limestone reliefs, where numerous springs and steep headwater streams converge to form three principal tributaries, Oued Mhajrat, Oued Khemis, and Oued Chekkoûr, which subsequently unite to form the Martil River, flowing through the urban and peri-urban areas of Tétouan and Martil before reaching the Mediterranean Sea (Bekkali et al. 2024).

The regional climate is typically Mediterranean, with pronounced seasonality. Precipitation occurs mainly between October and April, whereas the period from May to September is generally dry and warm. Average annual rainfall varies between approximately 500 and 750 mm, while mean annual air temperature ranges from 15 to 19°C (Salhi et al. 2019).

**Sampling Sites**

**Figure 1.**
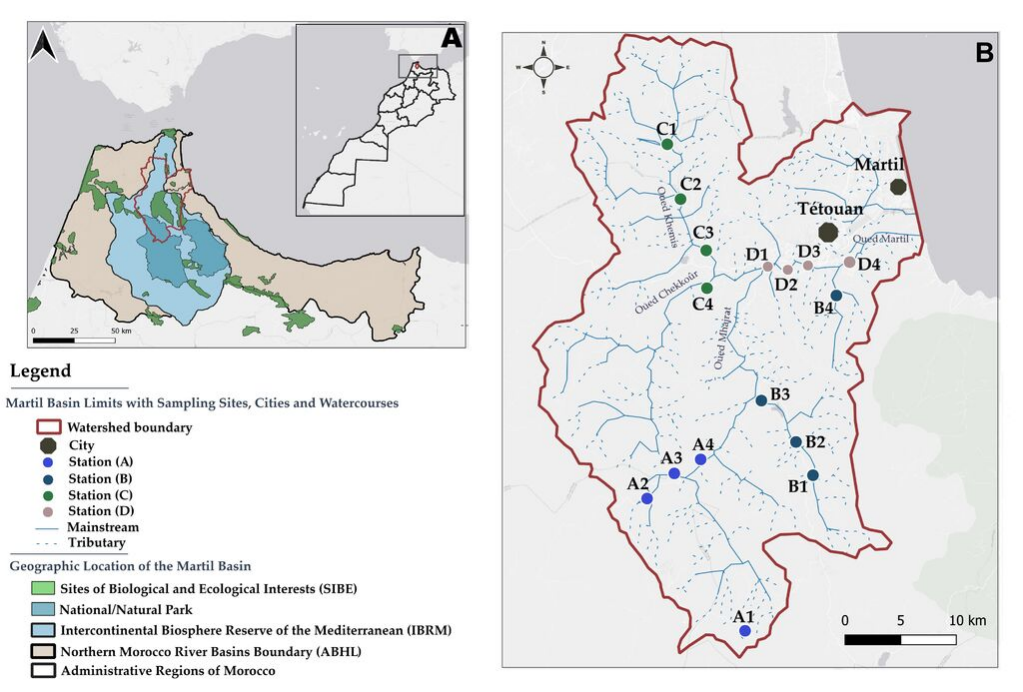
Geographical location of the Oued Martil Basin (northwestern Morocco) (A) and sampling stations (B).

The study was conducted at 16 sampling sites grouped into four categories (A–D) and distributed along an altitudinal gradient ranging from 5 to 1062 m across the main streams of the Martil basin, grouped into four categories: upstream natural and semi-natural sites (A), mid-stream semi-natural and agricultural sites (B), lower agricultural sites (C), and downstream urban-impacted sites (D). Sampling was performed during four seasonal campaigns: April 2017 (spring), early July 2017 (summer), November 2017 (autumn), and February 2018 (winter), encompassing the upstream–downstream continuum and covering the four principal hydrogeological zones of the basin (Fig. 1).

**Aquatic beetle sampling and identification**

Aquatic beetles were sampled seasonally using a kick-net (25 × 25 cm; 500 µm mesh) across all available microhabitats (riffles, pools, sandy, silty, and vegetated areas) with standardized effort (~45 min per site, minimum 5 min per microhabitat type), and continued within each habitat until repeated sweeps no longer yielded new taxa. Riffle sections were additionally sampled with a Surber sampler (20 × 20 cm) for presence/absence records only; Surber data were not pooled with kick-net data in quantitative analyses. All material was preserved in 96% ethanol for laboratory identification.

In the laboratory, only adult specimens were considered for identification and analysis. Specimens were examined under a binocular microscope and identified primarily to family or genus level using the taxonomic key of (Tachet et al. 2002), and subsequently identified to species level using specialized keys (Berthélemy et al. 1991, Castro and Delgado 1999, Gentili and Chiesa 1975, Jäch 1992, Jäch and Balke 2008, Olmi 1972, Schödl 1993. Identifications were performed with the assistance of N. Bennas, a specialist in aquatic Coleoptera and co-author of this study.

Furthermore, species were assigned to functional feeding groups (FFGs) according to the classifications proposed by Cummins (1974). In addition, functional habit groups (FHGs) were determined based on the criteria defined by Cummins (1974), Merritt and Cummins (1996).

**Physicochemical and hydrological analysis**

Physicochemical analyses were performed through in situ measurements of temperature (°C), dissolved oxygen (mg/L), electrical conductivity (μS/cm), and pH using a handheld multiparameter probe (EUTECH CyberScan PCD 650). Indicators of organic pollution, including Biochemical Oxygen Demand (BOD₅), Chemical Oxygen Demand (COD), and Total Suspended Solids (TSS), were analyzed with a Pastel UV multiparameter analyzer at the laboratory of the Loukkos Hydraulic Basin Agency (ABHL, Tétouan).

Hydromorphological characteristics, including water depth (cm), channel width (m), and flow velocity (m/s), were determined as the mean of three replicate measurements taken across the sampling area.

**Statistical analysis**

Spatiotemporal patterns in aquatic beetle assemblages and their relationships with environmental variables were visualized using boxplots, stacked bar, and bar charts produced with OriginPro 2024.

Descriptive statistics (mean and standard deviation) were calculated for physico-chemical, hydromorphological, and community metrics (species richness, total abundance). As normality assumptions were not achieved, the non-parametric Kruskal-Wallis test was applied to test relationships between environmental variables and community metrics using Spearman’s rank correlation.

A Factorial Correspondence Analysis (FCA) was performed on the species abundance matrix to investigate the seasonal structuring of beetle assemblages. To test for significant differences in community composition among the four habitat types (A–D), a Permutational Multivariate Analysis of Variance (PERMANOVA) was performed using the adonis2 function in the vegan package (Oksanen et al. 2025), based on a Jaccard dissimilarity matrix with 999 permutations. Where PERMANOVA indicated a significant effect, pairwise comparisons were conducted with the pairwise. adonis function, applying a Benjamini-Hochberg correction for multiple testing.

Community composition patterns were visualized using Non-Metric Multidimensional Scaling (NMDS) based on the same Jaccard dissimilarity matrix. The stability of the ordination was assessed via stress value.

Spatial heterogeneity in community composition was quantified by partitioning total β-diversity (βₛₒᵣ) into its turnover (βₛᵢₘ) and nestedness (βₛₙₑ) components using the Sørensen-based multiple-site dissimilarity framework implemented in the betapart package (Baselga and Orme 2012).

The relationships between aquatic beetle assemblages and environmental variables were examined using co-inertia analysis (CIA) in the ade4 R package (Dray and Dufour 2007). Species abundance data were Hellinger-transformed prior to analysis, and rare species (total abundance ≤ 1) were excluded to reduce ordination noise. Separate PCAs were performed on the biological (unscaled) and environmental (centered and scaled) matrices using dudi.pca, retaining the first two axes. Normality of environmental variables was assessed via the Shapiro-Wilk test; 8 of 11 variables deviated significantly from normality (p < 0.05), justifying the use of a Monte Carlo permutation test (999 permutations) to evaluate the global significance of the co-structure. All statistical analyses were performed in R Core Team 2024 (v4.4.2).

## Results

**Spatial distribution of beetle abundance and diversity**

**Figure 2.**
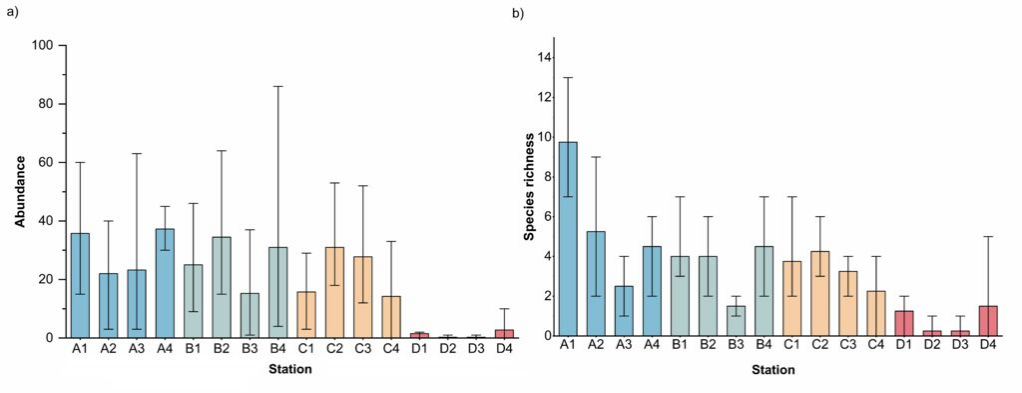
Longitudinal patterns of (A) species richness and (B) abundance of aquatic beetles in the Martil River Basin.

Examination of 1,268 aquatic beetles collected across 16 Martil basin sites yielded 50 species from 10 families and 27 genera. The site Tkaraa (A1), located in Bouhachem Natural Park, hosted the highest diversity with 22 species (13 in summer 2017), whereas the Zarka site (B4) yielded the highest abundance, with 194 individuals (86 in autumn 2017). Only a single aquatic beetle species occurred at heavily urbanized downstream sites such as Taboula (D2) and Roumana (D3), near Tetouan city (Fig. 2). Suppl. material 1 presents the list of species, their corresponding codes, functional feeding and habitat groups, and their occurrence across station groups.

**Seasonal variation in family composition**

With 607 individuals, Gyrinidae was the most abundant family, accounting for 54% of all aquatic beetles, followed by Elmidae with 262 individuals (20.6%), whereas the remaining families were far less numerous. However, Dytiscidae was the most diverse family in the study area, with 17 species, and *Agabus* was the most species-rich genus in the Martil River. *Gyrinus dejeani* (Brullé, 1832) and *Aulonogyrus striatus* (Fabricius, 1792) were the most widespread taxa, occurring at 12 of the 16 surveyed sites.

Seasonally, species richness peaked in spring 2017 with 31 species, which also supported the highest abundance (377 individuals), whereas post-flood winter 2018 yielded only 208 individuals from 19 species.

**Figure 3.**
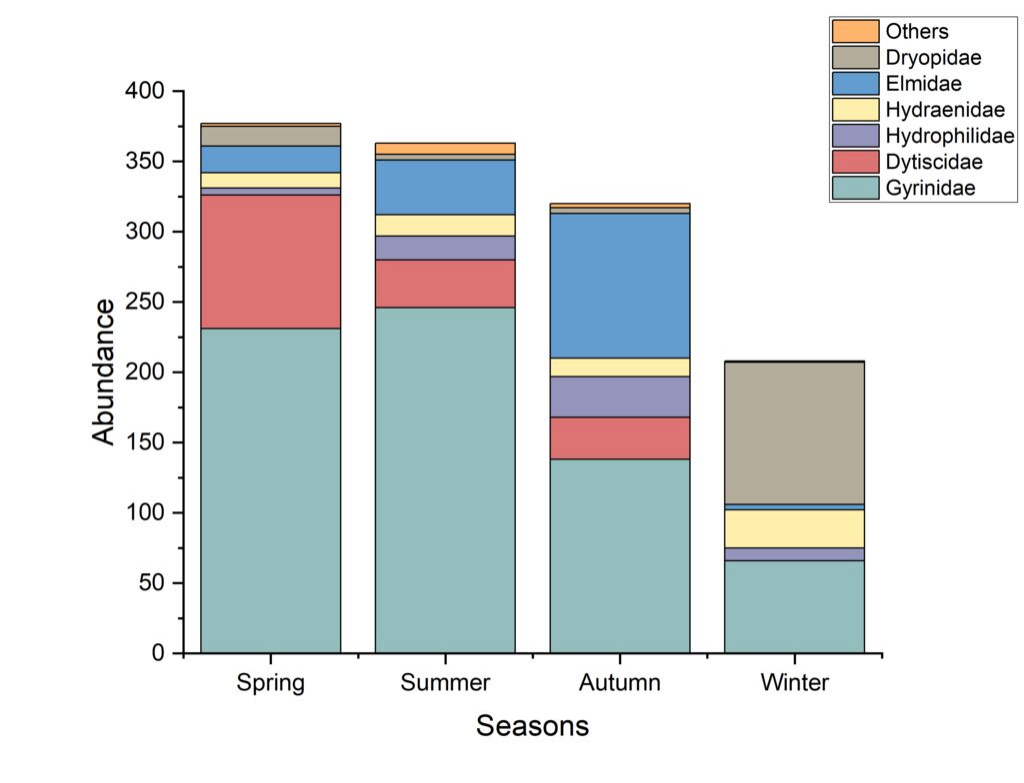
Stacked bar shows seasonal variation in the relative abundance of aquatic beetle families in the Martil basin.

Fig. 3 indicates that Gyrinidae dominate the assemblage, particularly during the dry period, when they are largely associated with lentic habitats. Dytiscidae are likewise abundant but reach their maximum representation in spring. In contrast, Elmidae occurred most frequently in autumn under sustained baseflow and rewetting conditions, whereas Dryopidae reached their highest relative contribution during high-flow winter periods.

**Seasonal contrasts between Adephaga and Polyphaga suborders**

**Figure 4.**
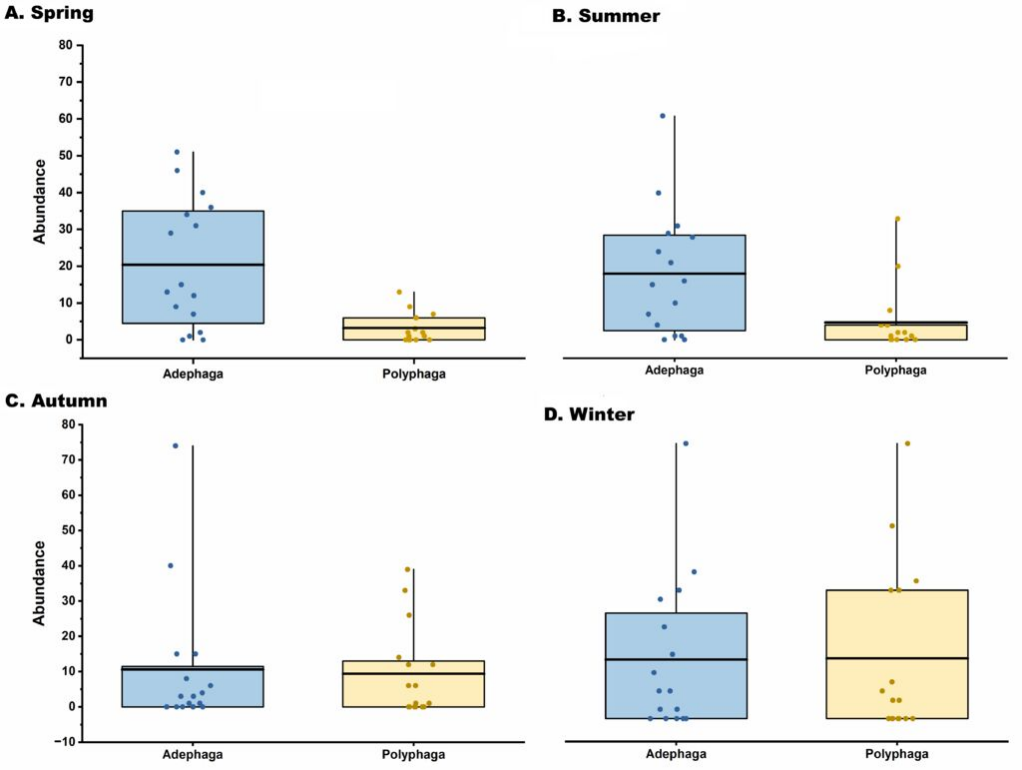
Box plots showing the seasonal variation in the abundance of the Adephaga and Polyphaga suborders across sampling stations in the Martil Basin: (A) spring, (B) summer, (C) autumn, and (D) winter.

Seasonal patterns revealed strong contrasts between the two beetle suborders. Adephaga (Gyrinidae, Haliplidae, Noteridae, Dytiscidae), which are primarily associated with lentic habitats, dominated during the dry period, with a mean abundance of 20.3 occurrences per station in spring (Fig. 4). In contrast, Polyphaga (Helophoridae, Hydrochidae, Hydrophilidae, Hydraenidae, Elmidae, Dryopidae), typically linked to lotic environments, were more frequent under wetter conditions. reaching an average of 9.38 occurrences per station in autumn. These seasonal contrasts likely reflect differences in habitat preference and dispersal capacity between the two suborders.

**Figure 5.**
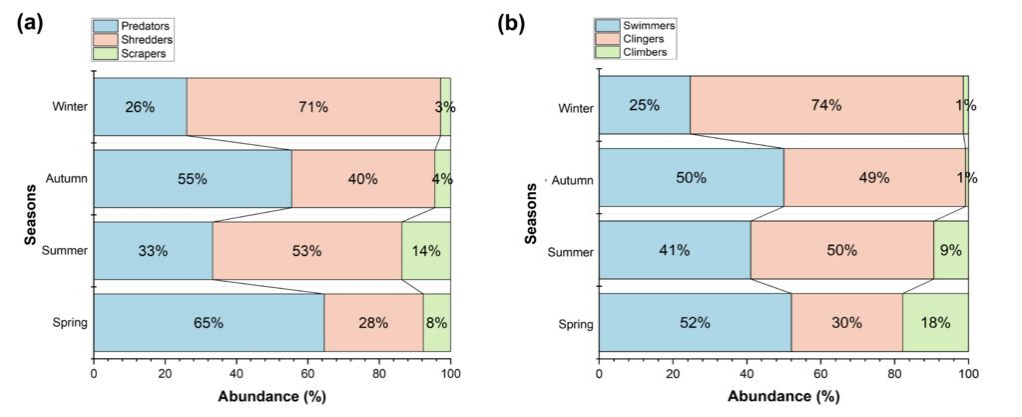
Seasonal distribution of (A) functional feeding, and (B) habit groups of aquatic beetles in the Martil basin.

Predators appeared to dominate the functional feeding groups, while swimmers constituted the major share of the functional habit profile. However, this overall pattern was strongly influenced by the overwhelming abundance of surface-dwelling Gyrinidae. Once this family was excluded from the analysis, shredders showed a clear preference for the winter period, whereas predators remained dominant only in spring (Fig. 5A). In parallel, the functional habit structure shifted toward a more benthic and substrate-associated assemblage, with clingers replacing swimmers as the dominant group during the wetter season, particularly in winter. Swimmers, in contrast, were more associated with stable hydrological conditions (Fig. 5B).

**Figure 6.**
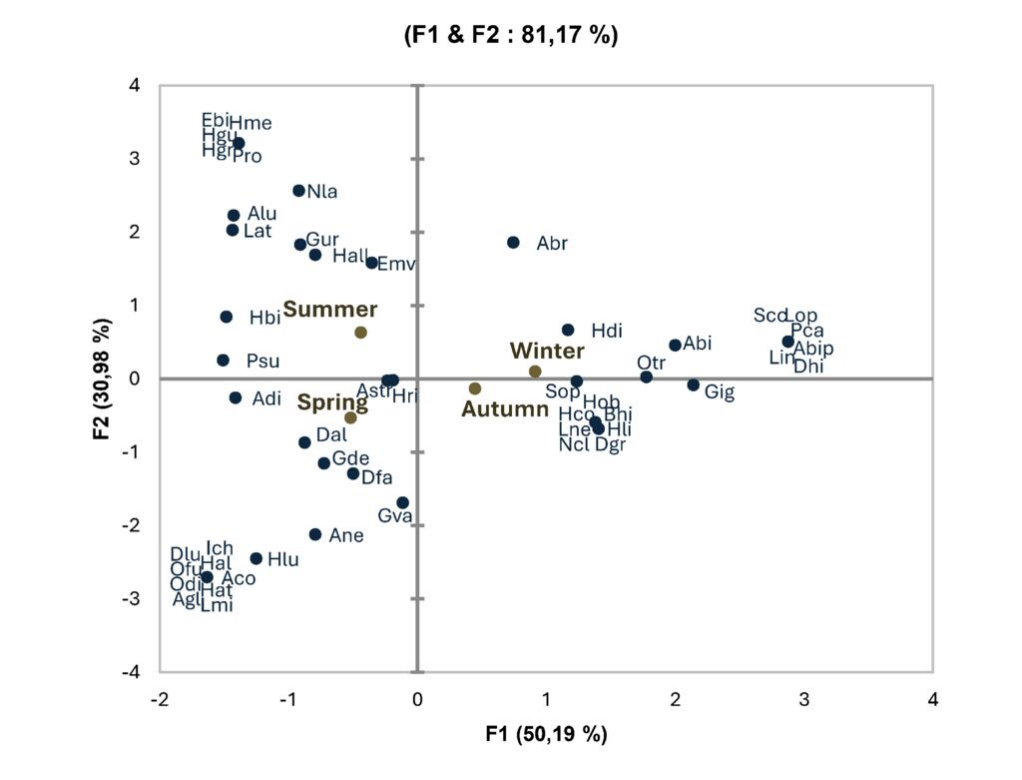
Factorial Correspondence Analysis (FCA) ordination of aquatic beetle assemblages across seasons in the Martil basin.

Factorial Correspondence Analysis (FCA) was used to explore the seasonal structuring of beetle assemblages in the Martil basin. The first two axes (CA1 and CA2) explained 50.19% and 30.98% of the total inertia, respectively, and revealed a clear segregation of species by season (Fig. 6).

**Group A**: plotted on the right side of the CA ordination. It comprises species such as *Agabus brunneus*, *Agabus bipustulata*, *Berosus hispanicus*, *Dryops gracilis*, *Deronectes hispanicus*, *Graptodytes ignotus*, *Hydroporus discretus*, *Haliplus lineatocollis*, *Hydroporus obsoletus*, *Limnius intermedius*, *Limnius neapolitanus*, *Limnius opacus*, *Nebrioporus clarkii*, *Oulimnius troglodytes*, *Peltodytes caesus*, *Stenelmis consobrina consobrina* and *Stenelmis optatus*, mainly associated with flowing conditions during the wet season.

**Group B**: included thermophilic taxa (*Anacaena lutescens*, *Enochrus bicolor*, *Elmis maugetii velutina*, *Gyrinus urinator*, *Hydraena allomorpha*, *Hydraena bisulcata*, *Hydrochus grandicollis*, *Hemisphaera guignoti*, *Hydroporus memnonius*, *Laccobius atrocephalus*, *Noterus laevis*, *Peltodytes rotundatus* and *Pomatinus substriatus*) that clustered toward the summer in the upper-left quadrant and were preferentially associated with calm, slow-flowing waters.

**Group C**: comprised eurythermic species (*Agabus conspersus*, *Agabus didymus*, *Anacaena globulus*, *Agabus nebulosus*, *Aulonogyrus striatus*, *Dryops algiricus*, *Deronectes fairmairei*, *Dryops lutulentus*, *Gyrinus dejeani*, *Graptodytes varius*, *Helophorus algiricus*, *Helophorus atlantis*, *Hydroporus lucasi*, *Hydraena rigua*, *Ilybius chalconatus*, *Laccophilus minutus*, *Ochthebius difficilis* and *Oulimnius fuscipes*), which showed a clear affinity for spring conditions characterized by relatively stable flow.

**Relationships Between Station Types and Beetle Species Distribution**

PERMANOVA results using Jaccard dissimilarity confirm that habitat type significantly structures aquatic assemblages (F = 2.04; df = 3, 12; R² = 0.337; P = 0.001; Table 1), accounting for 33.7% of the variation in community composition. This result highlights a compositional differentiation along the fluvial gradient, closely associated with contrasting land-use contexts.

Pairwise comparisons showed that urban assemblages diverged from upstream (p = 0.033), midstream (p = 0.030), and agricultural sites (p = 0.037), whereas differences among non-urban habitats were not statistically significant (all p > 0.05; Table 2). Together, these pairwise comparisons indicate a clear compositional break at urban downstream sites, whereas upstream, midstream, and agricultural reaches retain a more continuous assemblage structure along the river continuum.

**Figure 7.**
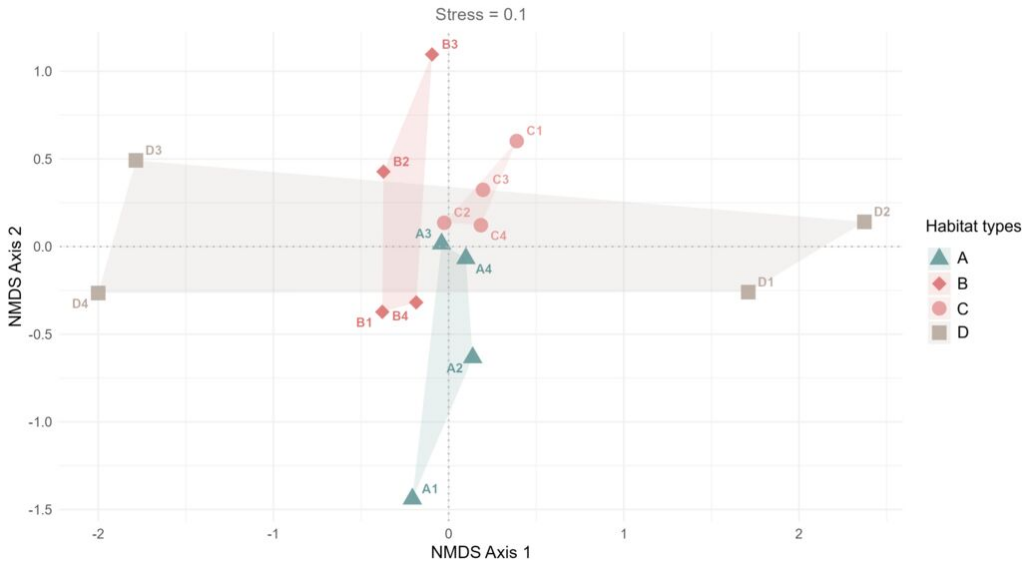
Non-metric multidimensional scaling (NMDS) ordination of aquatic beetle community composition across site groups (A–D) in the Martil River basin, based on Jaccard dissimilarity (stress = 0.100).

Non-metric multidimensional scaling (NMDS) ordination of aquatic beetle community composition based on Jaccard dissimilarities (stress = 0.100) indicates that the two-dimensional solution provides a reliable representation of compositional relationships among sites (Fig. 7). The ordination showed that urban sites tended to segregate from other habitat types, while upstream and midstream sites largely overlapped. Agricultural sites were positioned between these groups, reflecting an intermediate compositional status.

This spatial configuration is consistent with PERMANOVA results, confirming that urban downstream reaches host distinctly different assemblages, whereas non-urban habitats exhibit a more gradual compositional transition along the river.

**Figure 8.**
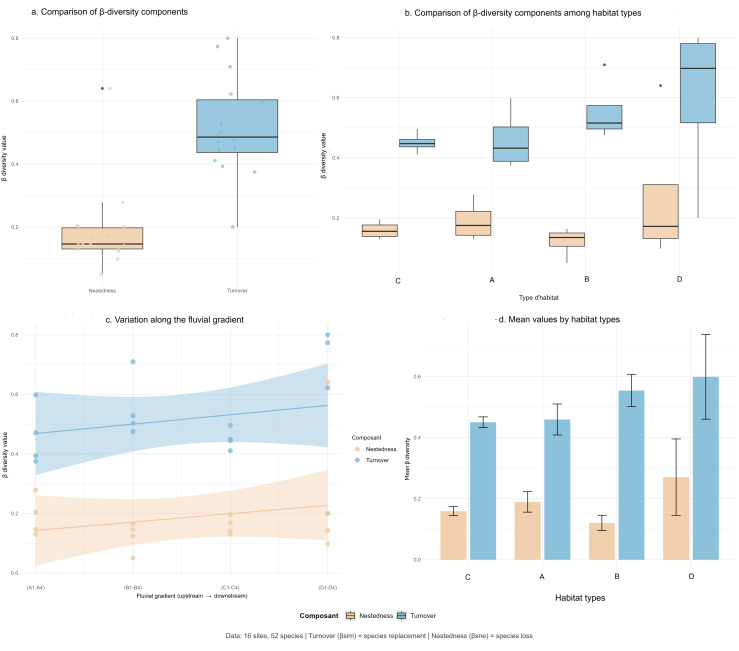
Components of β-diversity for aquatic beetle assemblages across habitat types (A–D) in the Martil River basin: (A) total β-diversity (βₛₒᵣ) and its partitioned components, turnover (βₛᵢₘ) and nestedness (βₛₙₑ), for all sampling sites; (B) mean (±SD) values of β-diversity components per habitat type; (C) relationship between β-diversity components and local species richness; (D) relationship between β-diversity components and longitudinal position along the river continuum.

Mean total β-diversity across sampling sites was high (β_sor_ = 0.701) (Fig. 8A), indicating pronounced spatial heterogeneity in aquatic Coleoptera assemblages along the studied river continuum. This spatial heterogeneity was predominantly driven by species turnover (β_sim_ = 0.516), which accounted for 73.7% of total β-diversity, whereas nestedness resultant dissimilarity contributed a substantially smaller proportion (β_sne_ = 0.185; 26.3%). Such a pattern underscores species replacement as the primary driver, suggesting strong environmental or spatial filtering mechanisms rather than simple richness-driven structuring.

Distinct β-diversity structures emerged among habitat types along the longitudinal gradient (Fig. 8B, C). Upstream sites exhibited the highest mean species richness (14.8 species) and a moderate level of total β-diversity (βₛₒᵣ = 0.649). Within these assemblages, turnover (βₛᵢₘ = 0.459) slightly exceeded nestedness (βₛₙₑ = 0.190). Midstream sites showed a marked reduction in species richness (mean = 8.0 species), accompanied by an increase in turnover (β_sim_ = 0.554) and the lowest nestedness values observed across the gradient (β_sne_ = 0.121). This configuration indicates assemblages structured predominantly by replacement processes, consistent with transitional environmental conditions along the longitudinal continuum. Agricultural downstream sites displayed intermediate richness levels (mean = 9.2 species) and the lowest total β-diversity among habitat types (β_sor_ = 0.610). In these sites, turnover (βₛᵢₘ = 0.450) and nestedness (βₛₙₑ = 0.159) contributed jointly to total β-diversity.

In contrast, urban downstream sites were characterized by extremely low species richness (mean = 2.5 species) but the highest total β-diversity (β_sor_ = 0.869). Both turnover (β_sim_ = 0.599) and nestedness (β_sne_ = 0.271) reached their highest mean values in these habitats.

Despite these contrasts in mean values among habitat types, statistical tests did not detect significant differences in turnover (Kruskal–Wallis χ² = 4.43; df = 3; P = 0.218) or nestedness (χ² = 2.47, p = 0.481). Likewise, no significant monotonic relationships were observed between β-diversity components and longitudinal position along the fluvial gradient (turnover: Spearman ρ = 0.267, p = 0.318; nestedness: ρ = 0.000, p = 1.000) (Fig. 8D). Spearman correlations between β-diversity components and local species richness were also non-significant (turnover: ρ = -0.286, p = 0.283; nested-ness: ρ = 0.232, p = 0.386).

Overall, these results indicate that spatial variation in aquatic Coleoptera assemblages is primarily structured by species turnover, with nestedness playing a secondary but non-negligible role, particularly in strongly disturbed urban downstream reaches.

**Correlations and longitudinal gradients in environmental conditions and beetle metrics**

Physicochemical conditions changed progressively from upstream (Group A) to downstream (Group D) sites, reflecting increasing anthropogenic influence (Table 3). Upstream sites (Group A) were characterized by favorable conditions, including higher dissolved oxygen and lower nutrient concentrations. In contrast, downstream reaches, particularly those designated as polluted (Group D), exhibited clear signs of organic enrichment and sediment loading. Mean water temperature increased from 15.5°C in Group A to 20.8°C in Group D. pH remained relatively stable across all groups, ranging between 7.3 and 7.8. However, electrical conductivity demonstrated a marked increase from 269 µS/cm upstream to 936 µS/cm at the most downstream sites.

A pronounced decline in dissolved oxygen was recorded, from 7.84 mg/L in Group A to 2.49 mg/L in Group D. Concurrently, the mean biological oxygen demand (BOD₅) reached 32.3 mg/L in Group D, compared to 10.7 mg/L in Group A. Similarly, chemical oxygen demand (COD) increased from 24.8 mg/L to 74.6 mg/L.

Hydromorphological alterations were evident along the gradient. While mean current velocity increased from 2.47 m/s to 8.02 m/s, water depth approximately doubled from 0.21 m to 0.41 m, and stream width expanded substantially from 4.93 m to 34.8 m.

Total abundance declined sharply from a mean of 29.6 individuals in Group A to 1.19 individuals in Group D. Species richness followed a similar trend, decreasing from 5.5 taxa upstream to 0.81 taxa at the most impacted downstream sites.

**Figure 9.**
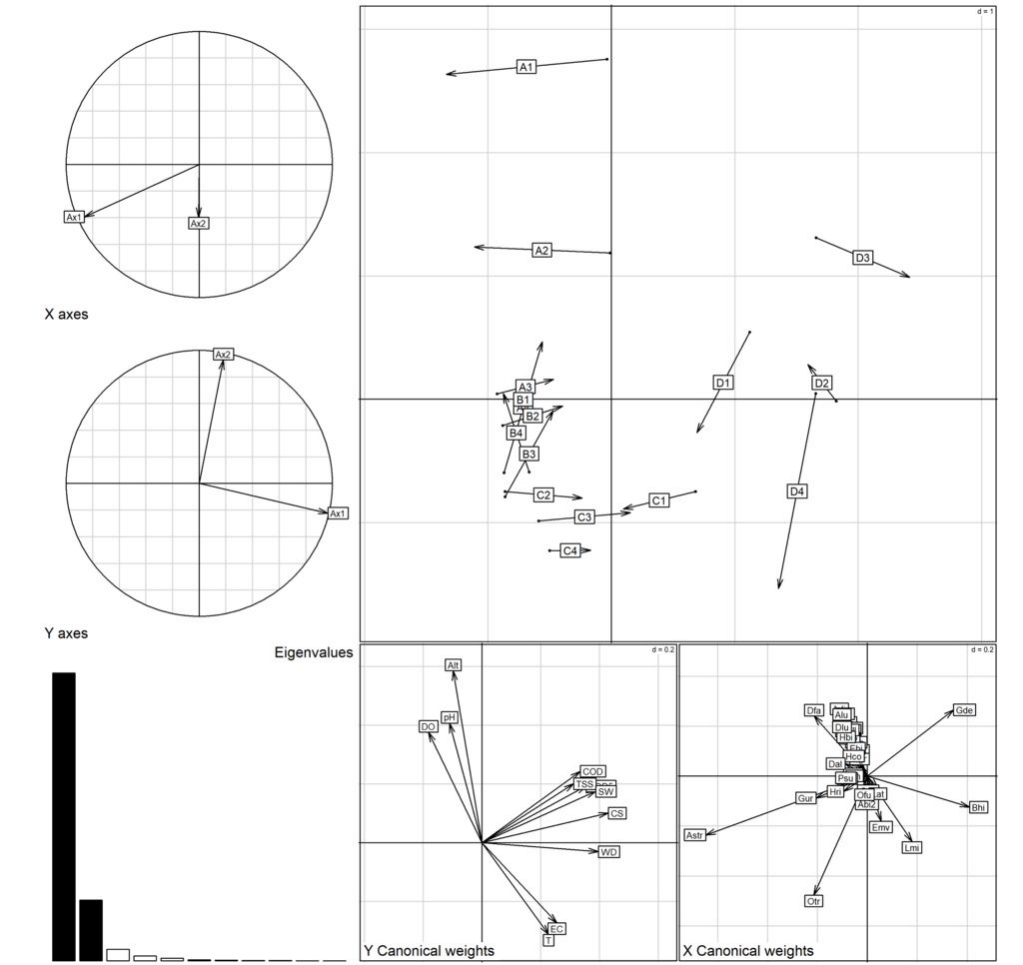
Co-inertia analysis (CIA) ordinations integrating relationships among environmental gradients, sampling stations, and beetle assemblages.

The co-inertia analysis (CIA) (Fig. 9) summarized the relationships between sampling sites, aquatic beetle assemblages, and environmental variables. The first two co-inertia axes accounted for 77.91% (axis 1) and 16.41% (axis 2) of the projected inertia, respectively, together explaining 94.32% of the total projected inertia of the joint structure. The global significance of the co-structure was evaluated using Monte Carlo permutation tests (999 permutations), which yielded a simulated P value of 0.004, indicating that the association between biological assemblages and environmental variables was statistically significant at the 5% threshold (RV = 0.467).

The ordination analysis reveals an altitudinal structuring of sampling sites along the first axis, consistent with environmental gradients. Sites from the upper natural areas are clustered in the upper portion of the positive side of Axis 2. The positive side of Axis 1 is primarily associated with downstream sites (Zone D). The upper part of the lower left quadrant of the biplot is occupied by sites from Zones A and B, which correspond to agricultural and semi-natural regions. The lower portion of this quadrant includes sites from the agricultural lower zone (Zone C).

Upstream sites in Zone A are characterized by rheophilic species such as *D. fairmairei* (Dfa), *H. discretus* (Hdi), *A. lutescens* (Alu), and *A. nebulosus* (Ane). This assemblage correlates strongly with high dissolved oxygen levels in the upper part of the ordination space. Agricultural sites supported intermediate assemblages dominated by moderately tolerant species responding to nutrient enrichment and elevated conductivity, such as *G. urinator* (Gur), *E. maugetii velutina* (Emv), and *O. fuscipes* (Ofu). Downstream Zone D sites are associated with species such as *L. minutus* (Lmi) and *H. atlantis* (Hat). Their presence reflects adaptations to slow-flowing, deeper habitats influenced by organic pollution (BOD_5_ and COD).

## Discussion

Aquatic Coleoptera assemblages along altitudinal and longitudinal gradients in the Martil basin are primarily shaped by environmental drivers such as altitude, slope, and current velocity. Seasonal ordination clearly separated taxa associated with wet-season lotic conditions from thermophilic species favored by warm summer pools throughout the year. Adephaga, particularly Gyrinidae and Dytiscidae, dominated during dry periods, reflecting their adaptation to lentic and warm habitats, while Polyphaga, including Elmidae and Dryopidae, were more abundant during wetter seasons, indicating a preference for lotic conditions.

The dominance of Gyrinidae, along with the high taxonomic diversity of Dytiscidae observed in our study, has been documented in several studies conducted in Morocco and across the Mediterranean region (Benetti et al. 2007, Belhaj et al. 2023b). These patterns suggest that gyrinids and Dytiscids function as successful generalist taxa, exhibiting strong adaptability to both hydroclimatic fluctuations and anthropogenic pressures.

The functional trait structure of aquatic beetle assemblages is characterized by a predominance of swimmers and predators, a pattern commonly observed in Mediterranean temporary waters, where seasonal drying favors taxa that combine active dispersal, flexible life-history strategies, and rapid recolonization (Touaylia 2017). Predatory beetles may additionally benefit from prey concentration in shrinking pools during low-water periods. Many dominant Adephaga are atmospheric breathers with sub-elytral air stores, enabling them to tolerate warm, oxygen-poor conditions in isolated pools. By contrast, Elmidae, Hydraenidae, and some Dryopidae rely on plastron or cuticular respiration, making them strongly dependent on well-oxygenated running water and therefore disadvantaged when flow ceases and dissolved oxygen declines during the dry season (Jooste et al. 2020, Guellaf et al. 2025b).

It is important to acknowledge that this functional signal is strongly influenced by Gyrinidae, which alone accounted for 54% of the total catch. Because gyrinids are classified as both swimmers and predators, their numerical dominance contributes substantially to the high proportions of these two functional groups.

Shifts in physicochemical and hydrological conditions, such as increased temperature, conductivity, organic enrichment, and sedimentation, were strongly associated with reduced habitat heterogeneity and declines in species richness and abundance, particularly in lowland reaches exposed to anthropogenic disturbances (Picazo et al. 2012, Valente-Neto et al. 2020).

Co-inertia analysis identified a primary gradient opposing high-altitude, well-oxygenated sites, hosting diverse rheophilic assemblages, to lowland, urban-influenced stations dominated by tolerant generalists adapted to warm, slow-flowing, and organically enriched waters. This pattern is consistent with observations from studies on altitude and flow intermittence affecting aquatic beetles and other macroinvertebrates throughout the Mediterranean region (Touaylia et al. 2013, Valente-Neto et al. 2020).

Our results indicate a pronounced compositional break between urban (D) and non-urban (A–C) sites, driven primarily by species turnover (βₛᵢₘ), where specialist-rich upstream assemblages are replaced by generalist-dominated communities, consistent with anthropogenic stress restructuring coleopteran assemblages (Aspin and House 2022). Along the longitudinal gradient, PERMANOVA, NMDS, and beta-diversity analyses revealed that upstream sites maintained high richness through species replacement, midstream sites showed replacement-dominated structure reflecting transitional hydrological conditions, agricultural sites combined replacement with moderate richness loss linked to homogenization and wet–dry seasonality, while urban sites exhibited both strong turnover and nestedness, indicating concurrent species loss and replacement.

Species with narrow distribution ranges were predominantly found in upper reaches and riffle habitats, whereas widely distributed species were more frequent in lower sites and stagnant habitats (Stanković et al. 2019). Adephaga species, known for their high dispersal and movement capacity, were widespread across all sampling stations. Stations A1 and A2, situated within the Bouhachem Natural Park and in permanent watercourses of B, displayed a high rate of endemism, particularly among Polyphaga species, highlighting their importance as critical refuges for these taxa.

However, *Helophorus atlantis* is a Moroccan endemic species classified as highly vulnerable (Benamar et al. 2021). In our dataset, it was recorded only in downstream Zone D, characterised by reduced flow and increased anthropogenic influence. In intermittent rivers, high-flow events can act as mass dispersal events, passively transporting upstream individuals into lowland reaches (Datry et al. 2014), and such records may therefore reflect transient trapped populations rather than truly tolerant residents. Whether this represents genuine ecological tolerance or episodic drift remains unclear, and further work is needed to better understand how Moroccan endemic aquatic beetles respond to environmental disturbance.

Most hotspots of water beetle richness and endemism in Morocco are concentrated in the humid northern mountain systems, notably the Rif, Middle Atlas, and northern Central Plateau (Belhaj et al. 2023a). However, conservation efforts in the Mediterranean region, including the Martil basin, remain insufficient, as current protected networks often overlook the full diversity of aquatic fauna, especially in midstream sectors where sensitive and endemic species are restricted to refuges in permanent watercourses (Nogueira et al. 2021, Bennas et al. 2009, Benamar et al. 2021). Expanding protection to permanent mid-reaches and reducing pollution and hydromorphological degradation in downstream areas would improve longitudinal connectivity and limit biotic homogenization, thus helping maintain diversity and support more resilient aquatic communities (Nogueira et al. 2021).

Water beetles serve as effective bioindicators for identifying priority conservation areas, and their integration into iterative complementarity approaches can efficiently guide the establishment of future protected aquatic sites (Dong et al. 2014, Bilton et al. 2019). However, with intensifying intermittency, many permanent watercourses in North Africa remain underrepresented in existing protected area networks, underscoring the urgent need for targeted conservation planning and robust, long-term monitoring programs to ensure the persistence of freshwater ecosystems in the region (Benamar et al. 2021, Belhaj et al. 2024, Guellaf et al. 2025a).

## Conclusions

This study reveals pronounced spatiotemporal structuring in aquatic beetle assemblages along the Martil River, driven by hydrological intermittency and anthropogenic gradients that limit endemic and sensitive species to upstream refugia while leaving only tolerant taxa downstream.

A notable pattern in our results is that pairwise PERMANOVA did not detect significant compositional differences among non-urban habitat types (A–C), These results may indicate that assemblages maintain a degree of compositional continuity under moderate agricultural pressure. In contrast, urban sites represent a distinct shift, consistent with a threshold response to severe pollution.

These patterns mirror broader Mediterranean dynamics, where urbanization and agriculture amplify intermittency, threatening endemism and diversity gradients. Expanding protected networks to permanent reaches, informed by beetle traits, emerges as a critical strategy to mitigate flow regime shifts and enhance resilience.

## Supplementary Material

2C2A7313-CE3F-5A8D-AE62-8E7EC5E1B37D 10.3897/BDJ.14.e192023.suppl1 Supplementary material 1 List of aquatic beetles recorded in the Martil basin during the survey period Data type Species distribution data Brief description The abundance of each taxon is indicated using the following codes: * = 1–10 individuals, ** = 10–100 individuals, *** = more than 100 individuals. Functional Feeding Groups (FFG) are indicated as Predators (Pr), Shredders (Sh), and Scrapers (Sc). Functional Habitat Groups (FHG) describe the main locomotor or substrate preference of taxa: Swimmers (Sw), Clingers (Cl), and Climbers (Cb). Chorotypes indicate the biogeographical distribution of each species: endemic to Morocco (Mor), Maghrebian (Mag), Ibero-Maghrebine (Ib-Mag), Mediterranean (Med), and species with a wide distribution (L-D). File: oo_1670370.xlsx https://binary.pensoft.net/file/1670370 Achraf Guellaf*1, Nard Bennas1, and Kawtar Kettani1

## Figures and Tables

**Table 1.** Results of PERMANOVA testing the effect of habitat type (groups A-D) on aquatic beetle community composition, based on Jaccard dissimilarity

**Analysis**	**Dependent variable**	**Factor**	**df**	**Sum of squares**	**F-value**	**R²**	**p-value**	**Significance**
PERMANOVA	Community composition	Habitat type	3	1.702	2.036	0.337	0.001	***
		Residual	12	3.344	-	0.663	-	-
		Total	15	5.046	-	1.000	-	-
Notes: *p < 0.05, **p < 0.01, ***p < 0.001, ns = not significant								

**Table 2.** Pairwise PERMANOVA comparisons between habitat types.

**Comparaison**		**R²**		**F-value**		***p* -value**		**Signifiance**	
A sites vs. D sites		0.290		2.858		0.033		*	
B sites vs. D sites		0.248		2.642		0.030		*	
C sites vs. D sites		0.293		2.907		0.037		*	
A sites vs. B sites		0.207		1.568		0.071		ns	
A sites vs. C sites		0.244		2.265		0.063		ns	
B sites vs. C sites		0.206		1.558		0.056		ns	
Notes:*p < 0.05, **p < 0.01, ***p < 0.001, ns = not significant; R² values represent the proportion of variance explained by habitat type in pairwise comparisons. All tests based on 999 permutations using Jaccard dissimilarity										

**Table 3.** Means ± SD of physicochemical and hydromorphological variables, abundance, and species richness of sampled aquatic beetles along the longitudinal gradient across station groups A, B, C, and D in the Martil Basin.

**Variables**	**A**	**B**	**C**	**D**
**Temperature (°C)**	15.5 ± 6.07	18.3 ± 6.29	19 ± 7.15	20.8 ± 6.60
**pH**	7.78 ± 0.45	7.73 ± 0.339	7.34 ± 0.334	7.51 ± 0.415
**Electrical conductivity (μS/cm)**	269 ± 147	397 ± 120	852 ± 245	936 ± 570
**Dissolved oxygen (mg/L)**	7.84 ± 4.76	5.63 ± 4.56	3.85 ± 2	2.49 ± 2.23
**Biological oxygen demand (mg/L)**	10.7 ± 6.24	9.09 ± 5.39	11.8 ± 7.62	32.3 ± 25.1
**Chemical oxygen demand (mg/L)**	24.8 ± 7.45	22 ± 10.5	25.7 ± 12.7	74.6 ± 73.3
**Suspended matter (mg/L)**	49.3 ± 37.7	33.6 ± 23.3	48 ± 31.6	121 ± 92.1
**Current Speed (m/s)**	2.47 ± 0.83	2.59 ± 0.634	3.65 ± 1.34	8.02 ± 2.48
**Water depth (m)**	0.21 ± 0.05	0.21 ± 0.03	0.293 ± 0.06	0.413 ± 0.09
**Stream width (m)**	4.93 ± 1.95	4.98 ± 1.11	4.47 ± 1.5	34.8 ± 18.2
**Abundance**	29.6 ± 19.3	26.4 ± 23.1	22.2 ± 15.2	1.19 ± 2.48
**Species richness**	5.5 ± 3.43	3.50 ± 2.07	3.38 ± 1.75	0.812 ± 1.33
